# Deep intronic variant causes aberrant splicing of *ATP7A* in a family with a variable occipital horn syndrome phenotype

**DOI:** 10.1016/j.ejmg.2023.104907

**Published:** 2024-02

**Authors:** J. Robert Harkness, Huw B. Thomas, Jill E. Urquhart, Peter Jamieson, Genomics England Research Consortium, Raymond T. O'Keefe, Helen M. Kingston, Charulata Deshpande, William G. Newman

**Affiliations:** a Manchester Centre for Genomic Medicine, Manchester University NHS Foundation Trust, Health Innovation Manchester, Manchester, UK; b Division of Evolution, Infection and Genomics, Faculty of Biology, Medicine and Health Sciences, University of Manchester, Manchester, UK

**Keywords:** ATP7A, Deep intronic variant, Non-coding, Splicing, Genome, Menkes disease, Occipital horn syndrome, Rare disease

## Abstract

Genetic variants in *ATP7A* are associated with a spectrum of X-linked disorders. In descending order of severity, these are Menkes disease, occipital horn syndrome, and X-linked distal spinal muscular atrophy. After 30 years of diagnostic investigation, we identified a deep intronic *ATP7A* variant in four males from a family affected to variable degrees by a predominantly skeletal phenotype, featuring bowing of long bones, elbow joints with restricted mobility which dislocate frequently, coarse curly hair, chronic diarrhoea, and motor coordination difficulties. Analysis of whole genome sequencing data from the Genomics England 100,000 Genomes Project following clinical re-evaluation identified a deep intronic *ATP7A* variant, which was predicted by SpliceAI to have a modest splicing effect. Using a mini-gene splicing assay, we determined that the intronic variant results in aberrant splicing. Sanger sequencing of patient cDNA revealed *ATP7A* transcripts with exon 5 skipping, or inclusion of a novel intron 4 pseudoexon. In both instances, frameshift leading to premature termination are predicted. Quantification of *ATP7A* mRNA transcripts using a qPCR assay indicated that the majority of transcripts (86.1 %) have non-canonical splicing, with 68.0 % featuring exon 5 skipping, and 18.1 % featuring the novel pseudoexon. We suggest that the variability of the phenotypes within the affected males results from the stochastic effects of splicing. This deep intronic variant, resulting in aberrant *ATP7A* splicing, expands the understanding of intronic variation on the *ATP7A*-related disease spectrum.

## 1 Introduction

Menkes disease (MIM 309400) is a rare X-linked copper metabolism disease caused by pathogenic variants in *ATP7A* (MIM 300011). Incidence rates of Menkes disease vary with conservative estimates suggesting 1 in 300,000 affected live births (Tønnesen et al., 1991; Vairo et al., 2019). *ATP7A* encodes the ubiquitously expressed Cu^2+^-transporting ATPase, a transmembrane protein which has dual functions as an ATP-dependent Cu^2+^ efflux channel, and a biomolecular loader of copper-dependent enzymes (cuproenzymes) in the trans-Golgi network (TGN) (Møller, 2015).

Menkes disease is the most clinically severe disease on an allelic spectrum of *ATP7A*-related disorders, which includes occipital horn syndrome (OHS; MIM 304150), and X-linked distal spinal muscular atrophy type 3 (SMAX3; MIM 300489) (Fradin et al., 2020; Zlatic et al., 2015). The severity of disease largely correlates to the deleteriousness of the causal *ATP7A* variant (Mhaske et al., 2020). Nonsense variants and large deletions correlate with an absence of functional protein, and cause Menkes disease, which manifest from the first few months of life. Menkes disease is primarily regarded as a neurodevelopmental disorder, featuring moderate to severe intellectual disability, epilepsy, and progressive neurodegeneration (Kaler et al., 1993; Rangarh and Kohli, 2018). Additionally, connective tissue anomalies, such as restricted bone growth, osteoporosis, and joint hyperflexibility, and ectodermal changes, including pili torti (kinky hair) and cutis laxa, and bladder diverticula are common from childhood. Patients may also experience chronic diarrhoea. If untreated, death usually occurs by three years of age. Early treatment with copper complexes such as copper-histidine offers the possibility to modify the onset of the severest phenotypes and extend life expectancy into adulthood (Vairo et al., 2019; Christodoulou et al., 1998; Nadal and Baerlocher, 1988; Tümer et al., 2017).

Less functionally deleterious variants such as missense variants and small inframe deletions, which permit residual ATP7A protein function, result in less-severe phenotypes associated with OHS (Møller, 2015; Dagenais et al., 2001). OHS features are similar to Menkes disease, but with absent or mild neurodegenerative changes, while non-neurological phenotypes can vary in severity (Kaler et al., 1993; Skjørringe et al., 2017). Typically described are occipital horns, which are wedge-shaped calcifications at the site of attachment of the trapezius and sternocleidomastoid muscles to the occipital bone (Gérard-Blanluet et al., 2004). Connective tissue anomalies are commonly described, alongside dysautonomia, which may cause chronic diarrhoea, syncope, and orthostatic hypotension. Early treatment of OHS patients with copper-histidine can reduce developmental anomalies associated with connective tissues to reduce the burden of disease (Vairo et al., 2019).

Finally, specific missense variants in the *ATP7A* gene are associated with late onset distal spinal muscular atrophy (SMAX3). This form of the disease features slowly progressive distal limb weakness and muscle wasting (Kaler et al., 1993), with occasional description of minor connective tissue anomalies (Gualandi et al., 2019). Although four different *ATP7A* variants (Gualandi et al., 2019; Kennerson et al., 2010; Shibuya et al., 2022) have been reported in cases of SMAX3, these all cluster in the terminal portion of the gene. Serum copper and caeruloplasmin are usually normal or slightly reduced in SMAX3 patients, suggesting that *ATP7A* variants causing SMAX3 are the mildest on the *ATP7A*-related phenotype spectrum (Fradin et al., 2020).

The function of ATP7A as a copper transporter is essential for copper dietary absorption via duodenal enterocytes into the portal circulation, and across the blood-brain barrier into the central nervous system (CNS) (Lutsenko et al., 2007; Maung et al., 2021). Inactivity of the ATP7A copper transporter therefore results in severe copper deficiency which particularly affects the brain, and accounts for the severest neurological phenotypes in Menkes disease (Zlatic et al., 2015). ATP7A-related copper deficiency impairs the activity of cuproenzymes, including cytochrome *c* oxidase (respiratory electron transport chain), and superoxide dismutase (maintenance of redox homeostasis), the dysfunction of which contribute to neurodegeneration identified in severely affected Menkes disease patients. At low copper concentrations, ATP7A localises to the trans-Golgi network (TGN), where it has a secondary function in the direct loading of copper to specific cuproenzymes. These cuproenzymes include lysyl oxidase (LOX) and lysyl oxidase-like (LOXL) proteins involved in collagen and elastin crosslinking (Trackman, 2018), copper-binding monooxygenases which contribute to catecholamine synthesis, and copper-dependent tyrosinases essential for biogenesis of signalling molecules such as melanin (Lutsenko et al., 2007). The reduced activity of LOX/LOXL proteins underlies the connective tissue manifestations of Menkes disease and OHS, such as cutis laxa and joint laxity (Horn and Wittung-Stafshede, 2021). Dysregulated trafficking of ATP7A between the TGN and the plasma membrane during high copper concentrations may also lead to dysfunction in copper homeostasis, rather than direct impairment of ATP7A copper transport (Skjørringe et al., 2017).

Here, we present four males from a single family affected by OHS with a novel deep intronic *ATP7A* variant, which leads to mis-splicing in a majority of *ATP7A* transcripts, leading to OHS. Phenotypes vary in severity between individuals, likely owing to the leaky effect of altered splicing.

### 1.1 Patient data

**Fig. 1:**
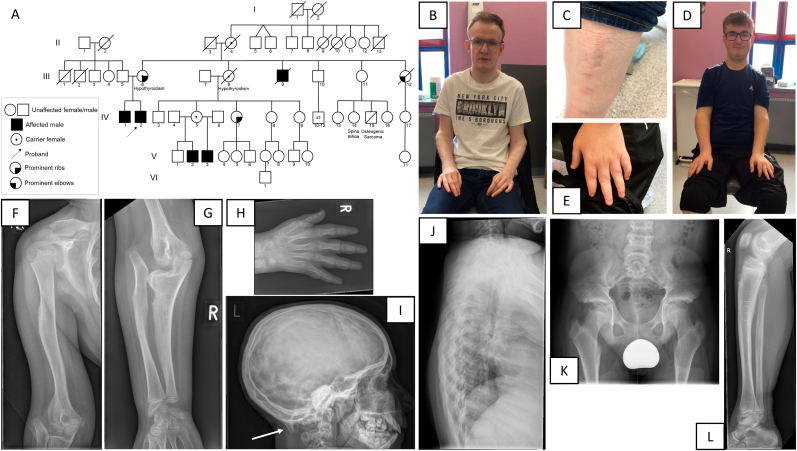
Family with X-linked *ATP7A*-related phenotype. (A) Pedigree of large family with five affected males (III:9, IV:1, IV:2, V:2 and V:3). (B–C) Photographs of V:2 indicating broad forehead with frontal prominences and prominent elbows unable to pronate/supinate (B) and cigarette paper scar on lower leg (C). (D–E) Photographs of V:3 indicating narrow thorax, and prominent elbows unable to pronate/supinate (D) and left hand indicating tapered fingers (E). (F–L) Full x-ray skeletal survey of V:3, including bowed right humerus (F), elbow with radiocapitellar dislocation and radius longer than ulna (G), hand with broad metacarpals and phalanges (H), lateral skull indicating occipital horns (arrow) (I), lateral spine showing scalloping of the vertebral bodies (J), pelvis with flattened acetabulae and broad iliac blades (K), and lateral view of anteriorly bowed tibia (L).

A three-week old male (Fig. 1A, IV:2) was referred to the paediatric clinic due to bilateral inguinal herniae. At the initial assessment, it was noted that he was failing to thrive and had diarrhoea. A sweat test performed at five weeks of age was reported as positive. His brother, IV:1 was assessed at the same time and in view of the similar history of chronic diarrhoea and failure to thrive, was investigated for cystic fibrosis. Both brothers had abnormal fecal elastase levels and although a molecular diagnosis of cystic fibrosis was not made, both patients responded to pancreatic enzyme replacement therapy. Their mother (III:6) had noticed that both boys had similar bony problems and had an unusual appearance of their elbows. Their maternal female cousin, IV:5, has two sons with similar appearances of elbows (V:2 and V:3, Fig. 1B–E) and a summary of the clinical features is presented in Table 1.

**Table 1:** Clinical features and investigations in four related males. ND, Not done.

Pedigree ID	IV1	IV2	V2	V3
Learning and development	Age appropriate early development Mild learning difficulty, currently employed	Mild delay in developmental milestones, mild learning difficulty	Walked 18 months, poor balance, needed additional support at school, currently employed	Age appropriate early development, poor fine motor skills, additional learning support at school, in vocational programme
Skeletal	Decidual teeth retained, winged scapulae, dislocated radial head, subluxed elbow, genu valgum requiring multiple surgeries	Winged scapulae, pectus carinatum, genu valgum, developing scoliosis, supernumerary teeth,dislocated radial head, subluxed elbow	Mild pectus excavatum, dislocated radial head, subluxed elbows, genu valgum - needed osteotomy	Plagiocephaly in infancy, narrow chest everted margins, genu valgum, dislocated radial head, subluxed elbow
Neurology	None	Hypotonia	Seizures - self limiting, limitation of dorsiflexion at ankles	Seizure like episodes in early childhood- self limiting
Ectodermal	Coarse hair, Inguinal hernia repair, poor healing and required revision, joint hypermobility, fingers and thumb sublux easily	Sparse, coarse hair, inguinal hernia repair at 3 weeks, recurred 6 years	Bilateral inguinal hernia repair	Inguinal hernia repaired in infancy, soft skin, joint hypermobility, poor skin healing, thin scars
Cardiology	Episodes of orthostatic hypotension - under investigation	None	None	None
Gastrointestinal	Diarrhoea from age 15 years	Frequent, pale offensive stools	Frequent stools	Frequent loose stools, post prandial hurry
Skeletal Survey	Narrow thorax, winged scapulae, bilateral radioular dislocation, delayed bone age	ND	ND	Occipital protruberance noted, bilateral radio-ulnar dislocaton with widening of ulna proximally, wide humeral diaphysis, broad metacarpals and phalanges, widening of interpenduclar distance in lumbar spine, posterior scalloping of vertebral
Other investigations	None	EMG/NCV – normal Muscle biopsy - normal	None	None

The possibility of an underlying skeletal dysplasia was considered in view of the appearance of their elbows, pectus excavatum in IV:2 and V:2, and a skeletal survey noting flared metaphyses and that the bone age was delayed by 2 years compared to chronological age for both boys. In view of the chronic diarrhoea and skeletal anomalies, an initial differential diagnosis of Schwachman-Diamond syndrome was considered, but was not confirmed by genetic testing. Full skeletal survey of V:3 (Fig. 1F-L) noted bowing of humerus and tibia, radiocapitellar dislocation of the elbow with radius longer than ulna, widening of the proximal ulna and widened diaphysis in the humerus radius and ulna. The metacarpals and phalanges appeared broad. Lateral x-ray of the skull noted presence of occipital horns. Scalloping of the vertebral bodies was present and pelvis featured flattened acetabulae and broad iliac blades.

IV:1 and IV:2 both remain on pancreatic enzyme replacement supplements. IV:1 has required revision of his inguinal herniae repair due to recurrence. Joint hypermobility impacts on his fine motor skills as interphalangeal joints lock and subluxate easily. There is a history of recurrent knee dislocation and all affected males have needed multiple surgery of the lower limbs. Notably, none of the affected males have occipital protuberances evident on clinical examination.

The maternal uncle of the proband (III:9) is deceased and did not pursue clinical assessment, so limited clinical details are available, but was reported to have similar problems with diarrhoea and with joint laxity to affected males in his family. Additionally, inability of affected males III:9, IV:1 and IV:2 to conceive a child has triggered questions from the family regarding effect of their disorder on fertility. Three females in the family (III:6, III:12, IV:7) also had prominent ribs or prominent elbows, traits which the family associates with the phenotypes in the affected males.

## 2 Methods

All participants provided informed written consent in accord with a protocol approved by the South Manchester Ethics committee (11/H1003/3, IRAS 64321). Genomics England has approval from the HRA Committee East of England – Cambridge South (REC Ref 14/EE/1112).

### 2.1 SNP array genotyping

SNP array analysis was performed with Genome-Wide Human SNP Array v.6.0 (Affymetrix) according to the manufacturer's protocol. Genotypes and copy number data were generated within the Affymetrix Genotyping Console (v.4.1.3.840) via the Birdseed V2 algorithm and SNP 6.0 CN/LOH algorithm, respectively.

### 2.2 Exome sequencing

Exome sequencing was carried out for one affected individual (IV:2) using the SureSelect Human All Exon Kit v4 (Agilent Technologies, Edinburgh UK) for the Illumina HiSeq 2500 system (Illumina, Cambridge, UK). Sequence data were mapped to the hg19 reference human genome using the Burrows–Wheeler aligner software (version 0.6.2; http://bio-bwa.sourceforge.net). Genome Analysis Tool Kit software (version 2.4.7; https://www.broadinstitute.org/gatk) was used for recalibration of base quality score and for indel realignment before using the unified genotyper (https://www.broadinstitute.org/gatk) for variant calling.

### 2.3 Whole genome sequencing

Whole genome sequencing (WGS) was conducted as part of the 100,000 Genomes Project (Genomics England). Blood samples, consent, clinical indication and HPO terms were collected for individuals III:6, IV:1, and IV:2 at their local hospital. Blood samples were sent to the Manchester regional genetic laboratory and DNA was extracted and sent to Illumina for WGS. Library preparation was performed using TruSeq DNA PCR-Free Library Prep, with 250 bp paired-end reads generated from the HiSeqX sequencer. Data were passed through Genomic England's bioinformatics pipeline. All variants within *ATP7A* locus (GRCh38:X:77910690-78050395) were filtered on the basis of quality control, allele frequency, and hypothesised allele segregation.

### 2.4 Sanger sequencing

Sanger sequencing was completed using Big Dye Terminator c.3.1 Cycle Sequencing Kit following manufacturer's instructions, and products were analysed with a 3730 Genetic Analyser instrument (Thermo Fisher Scientific). Primers for amplifying *ATP7A* intron 4 from genomic DNA were CTGTCACACATGGTGCATTG (forward) and GAGGGCAGATCGCTTGAGTC (reverse).

### 2.5 Minigene assay

A minigene vector was assembled to assess the effect of the intronic variant on splicing on upstream and downstream *ATP7A* exons. An SK3 vector was used, which is a derivative of the pSpliceExpress vector used previously (Thomas et al., 2020). A fragment of proband genomic *ATP7A* DNA, including the region from exon 4 to exon 5, plus 100 bp flanking sequence, was cloned into the SK3 minigene vector. Additionally, a DNA fragment was amplified from genomic DNA of the mother M1 was used to construct a WT ATP7A minigene vector for use as a control. The SK3 minigene backbone was isolated by digestion with restriction enzymes Nhe1 and BamH1. The genomic *ATP7A* DNA fragment was amplified by PCR using Phusion High-Fidelity DNA Polymerase (Thermo Fisher Scientific) using primers (forward gtacgggtgaccacgcgtccatgggTGGATTCTTTGCTACAATTATGATTTCATAAGTGCAT, reverse CCGGATCgagctgcatgtgtcagagGAATTTATTCAATTACATGCTACTAGATAGGAATCA) designed to produce fragments which overlap with the digested SK3 minigene backbone. The *ATP7A* fragment and the SK3 minigene backbone were assembled via the Gibson method and transformed into ultra-competent bacteria. Assembled *ATP7A* minigene vectors were isolated from bacterial colonies, and correct vector assembly was validated by Sanger sequencing performed by EuroFins Genomics.

HEK293 cells were cultured to 50% confluency in 2 ml of Dulbecco's modified Eagle's high-glucose medium (DMEMl Sigma-Aldrich), supplemented with 10 % foetal bovine serum (Sigma-Aldrich) in tissue-culture treated six-well plates at 37 °C with 5 % CO_2_. Cells were transfected with 1 μg of proband or maternal WT *ATP7A* minigene vector using Lipofectamine LTX reagent (Thermo Fisher Scientific) following manufacturer's instructions. Following 24 h incubation (37 °C, 5 % CO_2_), RNA was extracted from cells using TRI Reagent (Sigma-Aldrich) according to manufacturer's instructions. Extracted RNA was purified using RNeasy column clean-up kit (Qiagen), including the optional DNase digestion step. 5 μg of RNA was reverse transcribed to cDNA using Superscript Reverse Transcriptase (Thermo Fisher Scientific). cDNA was amplified by PCR using minigene specific primers (forward GCACCTTTGTGGTTCTCACT, reverse GGGCCTAGTTGCAGTAGTTCT), and products were run on 1% agarose gels supplemented with SafeView nucleic acid stain (NGS Biologicals). Gels were visualized using a blue-light transilluminator, and bands of interest were excised. DNA from excised fragments was extracted and purified using QIAquick gel extraction kit (Qiagen). Splicing products were confirmed by Sanger sequencing performed by EuroFins Genomics.

### 2.6 RNA extraction from patient cells

Patient derived lymphocyte cell line cultures, and fibroblast cultures were established by Specialised Cell Culture Services at St Mary's Hospital, Manchester following local procedures. Cells were harvested and centrifuged at 200×*g* for 5 min, and pellets were stored at −80 °C until RNA extraction. To extract RNA, cell pellets were thawed on ice prior to extraction using the Rneasy RNA extraction kit (Qiagen), following manufacturer's instructions, including the optional Dnase digestion step. 1 μg RNA was reverse transcribed to cDNA using High-Capacity RNA-to-cDNA kit (Applied Biosystems), following manufacturer's instructions. Patient cDNA was stored at −20 °C until further use.

### 2.7 Quantitative RT-PCR

Quantitative real time PCR was used to quantify exon junctions in the *ATP7A* transcript. Predesigned *ATP7A* probes Hs00921963_m1 (exon 1-2 boundary), Hs00921969_m1 (exon 4-5 boundary), and Hs00921970_m1 (exon 5-6 boundary) were used. A predesigned probe for *GAPDH* (Hs02786624_g1) was used as an endogenous control. All probes were 6-carboxy-fluorescein labelled and were purchased from ThermoFisher Scientific. 20 μl reactions containing cDNA, 20x FAM-labelled probe, and 2x TaqMan™ Gene Expression Master Mix (Applied Biosystems). PCR reactions were quantified using StepOnePlus™ Fast Real-Time PCR machine (Applied Biosystems). Standard curves of C_T_ values compared with log cDNA concentration were constructed by measuring tenfold serial dilutions of control cDNA from 100 to 0.01 ng/μl with each probe.

## 3 Results

### 3.1 Genetic analysis

**Fig. 2:**
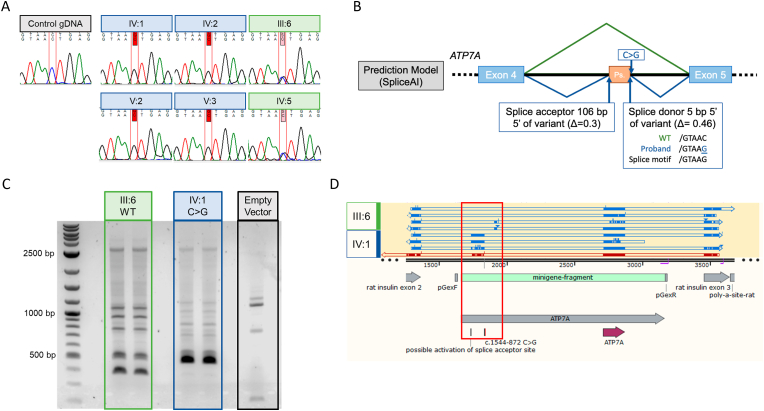
Evaluation of splicing changes from the *ATP7A* c.1544-872C>G intronic variant. (A) Sanger sequencing showing *ATP7A* c.1544-872C>G variant in affected individuals, which confirm the expected X-linked segregation. (B) Diagram of SpliceAI *in silico* splice prediction. The C>G variant strengthens +5 site in a GT-AG splice consensus motif. (C) Image of minigene assay reverse transcription product gel electrophoresis indicating larger transcripts are generated from the vector with the *ATP7A* c.1544-872C>G variant. (D) Alignment of minigene product sequencing alignment using SnapGene software, red box highlights alignment of pseudoexon in an assay with the *ATP7A* c.1544-872C>G variant. (For interpretation of the references to colour in this figure legend, the reader is referred to the Web version of this article.)

Linkage analysis of Affymetrix SNP array data from IV:1 and V:2 revealed three shared X-chromosome regions totaling 25.6 Mb (GRCh37 X:2,694,240-6,691,422 at Xp22.33-p22.31; X:53,887,456-58,107,416 at Xp11.22-p11.21 and X:69,445,677-86,811,737 at Xq13.1-q21.3). This linkage was consistent with the hypothesised X-linked transmission apparent in the family pedigree (Fig. 1A). No copy number variants of note were identified. Exome sequence analysis of IV:1 revealed a number of rare coding variants within the linked regions, but no compelling candidate (Supplemental data). Alongside his affected brother (IV:2), and their mother (III:6), IV:1 was recruited to the 100,000 Genomes Project for whole genome sequencing (WGS) analysis. Review of the clinical phenotype at this time noted the wiry hair and the possibility of an *ATP7A* related phenotype. Rare (allele frequency <0.01) coding and noncoding variants present at the *ATP7A* locus in the three individuals were filtered according to the expected X-linked mode of inheritance. One deep intronic *ATP7A* variant c.1544-872C>G (GRCh38 X:78002201C>G), within the previously linked region, was identified. Segregation analysis by Sanger sequencing (Fig. 2A) confirmed hemizygosity of the c.1544-872C>G variant among the four affected males for whom samples were available (IV:1, IV:2, V2, and V3), and heterozygosity in their respective mothers (III:6 and IV:5).

The effect of the variant on splicing was modelled using SpliceAI (Yasmeen et al., 2014). The C>G variant strengthens the +5 site within a consensus GT-AG splice donor motif (from GTAAC to GTAAG), resulting in increased probability of use by splicing machinery (Fig. 2B). This change is also predicted to strengthen activity of a cryptic acceptor site 106 bp upstream of the variant, suggesting the splicing of a novel pseudoexon in *ATP7A*.

### 3.2 Intronic *ATP7A* variant results in altered splicing

Minigene assays were performed, as described previously (Thomas et al., 2020), to determine the effect of the intronic variant on splicing activity. Minigene vectors were constructed with an *ATP7A* fragment from IV:1 genomic DNA, or the wild type (WT) allele from the proband's mother (IV:6). The *ATP7A* fragment contained the region from exon 4 to exon 5 of *ATP7A*, plus 200 bp flanking sequence. HEK293 cells were transfected with minigene-*ATP7A* vectors and RNA was extracted. Evaluation of reverse transcribe minigene assay products via gel electrophoresis indicated that proband *ATP7A*-minigene vector transcription products were larger compared to the WT vector (Fig. 2C). Sanger sequencing of these products confirmed the inclusion of the predicted novel ∼100 bp pseudoexon (Fig. 2D).

**Fig. 3:**
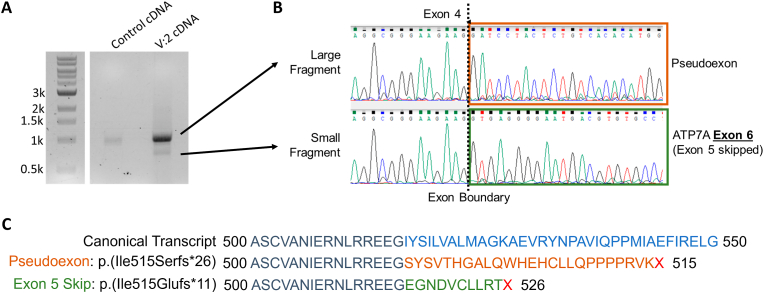
Analysis of *ATP7A* transcripts from V:2 lymphocyte-derived reverse transcribed RNA. (A) Electrophoresis of products from PCR of *ATP7A* exon 4-6 in IV:2 cDNA indicates two products are amplified. (B) Sanger sequencing traces of two PCR products from V:2. (C) Predicted effect of splicing changes on *ATP7A* translation products.

To validate these splicing changes, RNA was extracted from blood lymphocytes derived from V:2. RNA was reverse transcribed and exon 4-6 of *ATP7A* cDNA was sequenced to confirm presence of the novel *ATP7A* pseudoexon. We identified transcription products of two sizes, indicating the novel splice variant has a “leaky” effect (Fig. 3A). The larger fragment contained the predicted pseudoexon between exons 4 and 5. The smaller fragment did not contain the pseudoexon, but indicated skipping of exon 5 (Fig. 3B). Inclusion of the novel pseudoexon is predicted to create a frameshift p.(Ile515Serfs*26). Similarly, skipping of exon 5 is predicted to create a different frameshift p.(Ile515Glufs*11) (Fig. 3C). In both cases of mis-splicing, premature termination at residue 526 or 541 respectively is predicted to result in a truncated ATP7A protein consisting of the N-terminal cytoplasmic domain containing copper-binding motifs (aa1-653) and lacking the transmembrane (aa654-1406) and C-terminal cytoplasmic (aa1407-1500) domains. These truncated products are therefore predicted either to produce non-functional protein for copper transportation across plasma membranes, or be targeted for nonsense-mediated decay.

**Fig. 4:**
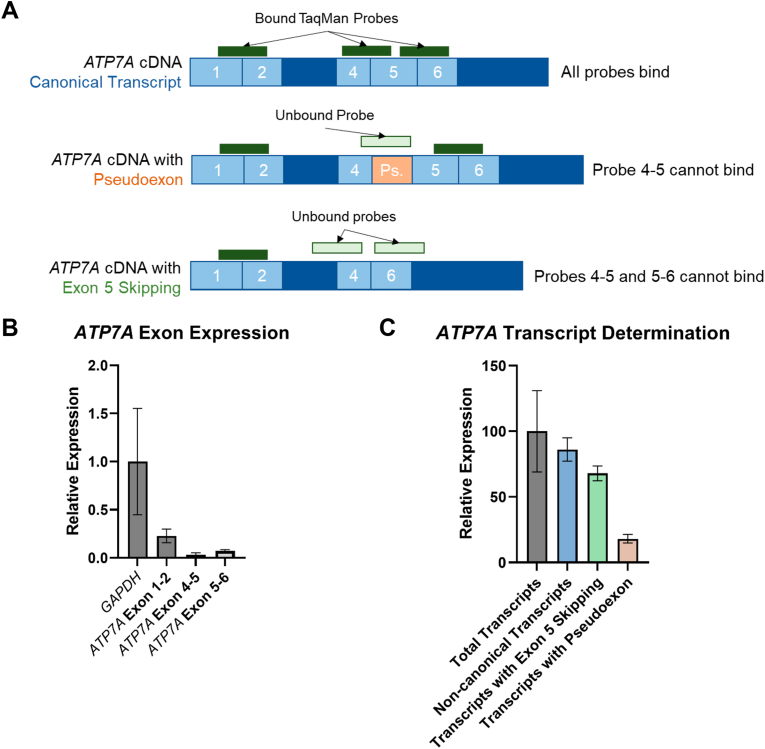
Quantitative PCR of *ATP7A* transcripts from V:2 lymphocyte cDNA. (A) Design of assay using three TaqMan probes specific to *ATP7A* exon boundaries to quantify proportions of transcripts affected by splicing. (B) QPCR of *ATP7A* exons in V:2 lymphocyte cDNA, relative to *GAPDH*. (C) Determination of proportion of *ATP7A* transcripts with pseudoexon and exon 5 skipping relative to total *ATP7A* transcripts. *ATP7A* and *GAPDH* expression was quantified from a healthy control using a standard curve by plotting log cDNA concentration against Ct, in two experiments each with three reactions.

In order to establish the proportion of total *ATP7A* transcripts disrupted by splicing, we performed quantitative PCR. TaqMan probes specific to *ATP7A* cDNA exon boundaries 1-2, 4-5, and 5-6 were used to quantify the relative proportion of transcripts which contained either the novel pseudoexon, or exon 5 skipping. Probes binding across exon boundaries at exon 4-5 and exon 5-6 were compared to total *ATP7A* transcripts (exon 1-2 boundary) to quantify the relative proportion of transcripts affected by mis-splicing (Fig. 4A). In V:2 lymphocyte derived RNA, qPCR analysis determined 13.9 % of all *ATP7A* transcripts were canonical, with 68.0 % of transcripts featuring exon 5 skipping and 18.1 % transcripts featuring the modelled pseudoexon between exons 4 and 5 (Fig. 4B and C). This analysis suggests the majority of *ATP7A* transcripts in lymphocyte-derived RNA have splicing altered by the *ATP7A* c.1544-872C>G variant, which is likely to reduce global *ATP7A* function in the hemizygous males.

## 4 Discussion

Here, we describe four males with a hemizygous novel intronic variant in *ATP7A*. Despite the variable phenotypes the four males with normal intellect are each affected by dislocated elbows, genu valgum, soft stretchy and poor healing skin with papyraceous scars and wiry, coarse hair. Some of the males also have a narrow thorax, inguinal herniae, keratosis pilaris, pectus excavatum, and chronic diarrhoea. These clinical features, plus biochemical evidence of low serum copper and caeruloplasmin levels, are consistent with a diagnosis of OHS. Notably, pancreatic insufficiency is not described as a feature of the *ATP7A*-related disease spectrum. No alternative explanation has been determined for this feature and should be screened for in other individuals determined to have *ATP7A*-related disease. Furthermore, self-reported inability of affected males to conceive children resulted in speculation of *ATP7A*-related copper homeostasis dysfunction affecting fertility. While there is no report of male infertility in *ATP7A*-related disease literature, retrograde ejaculation is a known symptom of dysautonomia associated with deficiency of dopamine-beta-hydroxylase (DBH), a copper dependent enzyme (Vincent and Robertson, 2002).

The majority of variants identified in *ATP7A* have been identified in patients with Menkes disease. Following variant classification analysis of *ATP7A* variants by Mhaske et al. (2020), of the pathogenic and likely pathogenic variants in *ATP7A,* 89 % were identified in patients with Menkes disease, with just 4 % in OHS (Mhaske et al., 2020). However, in reports from a single Danish center, there are similar proportions of patients with missense, nonsense, indel, and splice site variants in *ATP7A* (Møller, 2015). The difference in frequency between Menkes disease patients and nonsense variants associated with Menkes disease likely results from a clinical ascertainment bias in favour of the more severe and early onset features associated with Menkes disease. The less severe progressive natural history associated with OHS is therefore likely underdiagnosed, possibly due to lack of RNA-based analysis to detect variants which cause splicing defects in patients with milder phenotypes. We have identified a family affected by OHS with a deep intronic variant in *ATP7A*. This intronic variant introduces a novel splicing donor site and results in mis-splicing of a majority of *ATP7A* transcripts, either by skipping exon 5 or by inclusion of a novel pseudoexon. In both cases, transcripts are predicted to cause frameshift likely results in nonsense mediated decay.

The first patients to receive a molecular diagnosis of OHS due to deep intronic *ATP7A* variants were identified in a cohort in Denmark (Yasmeen et al., 2014). Intronic variants in intron 10 (c.2406 + 1117A>G), intron 14 (c.2916 + 2480T>G) and intron 16 (c.2916 + 2480T>G) were identified by performing overlapping RT-PCR reactions of the entire cDNA *ATP7A* sequence*.* In each case, the novel intronic variant led to inclusion of a pseudoexon into *ATP7A* transcripts and creation of a premature stop codon. Yasmeen et al. (2014) proposed that severity of phenotypes correlated with the relative proportion of transcripts containing the pseudoexon (Yasmeen et al., 2014). In the most severely affected Menkes disease patient, just 0.2 % of *ATP7A* transcripts were canonical. The proportions of canonical transcripts in the less severely affected patients were 11 % and 2 %, suggesting that the proportion of canonical transcripts required to prevent the onset of the severest phenotypes lies below a 2 % threshold. This hypothesis was supported by the subsequent investigation of five patients with splice-site variants leading to exon skipping (Møller et al.). In one patient with Menkes disease, 0.5 % of transcripts were canonical, which increased to 3.5 % canonical transcripts in patents with OHS. These findings suggest the relative strength of the noncoding variant resulting in splicing changes is important in predicting phenotypic severity.

Analysis of RNA from V:2 showed a higher proportion of canonical *ATP7A* transcripts (13.9 %) compared to the cases reported previously (Yasmeen et al., 2014; Møller et al.), with the majority of the non-canonical transcripts featuring exon 5 skipping, rather than pseudoexon inclusion, as a result of the *ATP7A* c.1544-872C>G variant. The milder *ATP7A*-related phenotype in the affected males presented here is consistent with this higher level of the canonical transcript. The analysis of transcripts from lymphocyte-derived RNA is one limitation of our analysis, as lymphocyte expression of *ATP7A* is lower compared to other cell types more relevant to the connective tissue phenotype, such as dermal fibroblasts.

The phenotypic variability of patients analysed here can be attributed to the inherent leakiness of splicing, a feature of the stochastic variation resulting from competition between acceptor sites of the pseudoexon and the downstream exon in affinity for binding of the spliceosome (Anna and Monika, 2018; Chin et al., 2022). This phenomenon has been characterized previously in other diseases, such as disease severity in cystic fibrosis in patients correlating with the degree of intron 19 pseudoexon inclusion resulting from the *CFTR* c.3718-2477C>T variant (Sobczyńska-Tomaszewska et al., 2013). The family reported here have a progressive disease, with onset in early childhood. Changes in the proportion of transcripts affected by mis-splicing in different tissue types over time may account for differences in onset and phenotypic variability in this family. Additionally, the phenotypic differences observed in our patients are biased by age at clinical assessment. The oldest pair of brothers (IV:1 and IV:2) are mature adults, while their cousins (V:2 and V:3) are young adults. The younger patients may develop additional or progressive phenotypes as observed in the older pair of brothers. Age of OHS onset ranges from early childhood to early adulthood, and phenotypes are usually progressive (Kaler et al., 1993). Discussion of phenotypic variability between these individuals would be better informed by tracking phenotype progression over time.

Finally, it is important to consider that there are factors aside from splicing which may affect phenotype severity. Previously, analysis of an *ATP7A* missense variant identified in brothers with variable phenotypes indicated that 17 % of wild type activity was maintained (Donsante et al., 2007). Variability in phenotype between the brothers was attributed to greater upregulation of *ATP7A* transcription in fibroblasts from the more mildly affected brother with the OHS phenotype, compared to his more severely affected brother with a Menkes syndrome phenotype. The authors suggested that the increased *ATP7A* expression compensated for lost ATP7A channel function, and that differences in overexpression underlay the phenotypic variability (Donsante et al., 2007). Factors influencing *ATP7A* expression may also facilitate the variation in phenotypes of the family reported here.

The identification of this family with a novel deep intronic *ATP7A* variant contributes to the understanding of OHS as a variable disease and illustrates the value in conducting WGS to identify deep intronic variants. Future work should establish how leaky splicing variants can lead to life-long variability in disease severity in different tissues to better understand disease progression and improve clinical management. Finally, studies should be undertaken to improve understanding of associations between *ATP7A*-related disease and male fertility.

## Funding

The research was funded through support from the 10.13039/100014653NIHR Manchester Biomedical Research Centre (IS-BRC-1215-20,007 and NIHR203308) to WGN and ROK and the 10.13039/100010269Wellcome Trust (PhD studentship to JRH).

## Ethical approval

All participants provided informed written consent in accord with a protocol approved by the South Manchester Ethics committee (11/H1003/3, IRAS 64321). Genomics England has approval from the HRA Committee East of England – Cambridge South (REC Ref 14/EE/1112).

## CRediT authorship contribution statement

**J. Robert Harkness:** Writing – review & editing, Writing – original draft, Visualization, Validation, Methodology, Investigation, Formal analysis, Data curation. **Huw B. Thomas:** Methodology, Conceptualization. **Jill E. Urquhart:** Investigation. **Peter Jamieson:** Formal analysis, Data curation. **Raymond T. O'Keefe:** Writing – review & editing, Supervision, Conceptualization. **Helen M. Kingston:** Writing – review & editing, Investigation, Data curation, Conceptualization. **Charulata Deshpande:** Writing – review & editing, Investigation, Data curation, Conceptualization. **William G. Newman:** Writing – review & editing, Supervision, Resources, Project administration, Conceptualization.

## Data Availability

Data will be made available on request.

## References

[bib1] Anna A., Monika G. (2018). Splicing mutations in human genetic disorders: examples, detection, and confirmation. J. Appl. Genet..

[bib2] Chin H.L., Lin S., Dalmann J., Modi B., Alderman E., Salman A. (2022). Can leaky splicing and evasion of premature termination codon surveillance contribute to the phenotypic variability in Alkuraya-Kucinskas syndrome?. Eur. J. Med. Genet..

[bib3] Christodoulou J., Danks D.M., Sarkar B., Baerlocher K.E., Casey R., Horn N. (1998). Early treatment of Menkes disease with parenteral Cooper-Histidine: long-term follow-up of four treated patients. Am. J. Med. Genet..

[bib4] Dagenais S.L., Adam A.N., Innis J.W., Glover T.W. (2001). A novel frameshift mutation in exon 23 of ATP7A (MNK) results in occipital horn syndrome and not in Menkes disease. Am. J. Hum. Genet..

[bib5] Donsante A., Tang J., Godwin S.C., Holmes C.S., Goldstein D.S., Bassuk A. (2007). Differences in ATP7A gene expression underlie intrafamilial variability in Menkes disease/occipital horn syndrome. J. Med. Genet..

[bib6] Fradin M., Lavillaureix A., Jaillard S., Quelin C., Sauleau P., Minot M.C. (2020). ATP7A mutation with occipital horns and distal motor neuropathy: a continuum. Eur. J. Med. Genet..

[bib7] Gérard-Blanluet M., Birk-Møller L., Caubel I., Gélot A., Billette de Villemeur T., Horn N. (2004). Early development of occipital horns in a classical Menkes patient. Am. J. Med. Genet..

[bib8] Gualandi F., Sette E., Fortunato F., Bigoni S., De Grandis D., Scotton C. (2019). Report of a novel ATP7A mutation causing distal motor neuropathy. Neuromuscul. Disord..

[bib9] Horn N., Wittung-Stafshede P. (2021). ATP7A-Regulated enzyme metalation and trafficking in the Menkes disease puzzle. Biomedicines.

[bib10] Kaler S.G., DiStasio A.T., Adam M.P., Ardinger H.H., Pagon R.A., Wallace S.E., Bean L.J., Gripp K.W. (1993). GeneReviews® [Internet].

[bib11] Kennerson M.L., Nicholson G.A., Kaler S.G., Kowalski B., Mercer J.F.B., Tang J. (2010). Missense mutations in the copper transporter gene ATP7A cause X-linked distal hereditary motor neuropathy. Am. J. Hum. Genet..

[bib12] Lutsenko S., Barnes N.L., Bartee M.Y., Dmitriev O.Y. (2007). Function and regulation of human copper-transporting ATPases. Physiol. Rev..

[bib13] Maung M.T., Carlson A., Olea-Flores M., Elkhadragy L., Schachtschneider K.M., Navarro-Tito N. (2021). The molecular and cellular basis of copper dysregulation and its relationship with human pathologies. FASEB J. Off. Publ. Fed. Am. Soc. Exp. Biol..

[bib14] Mhaske A., Dileep K.V., Kumar M., Poojary M., Pandhare K., Zhang K.Y.J. (2020). ATP7A Clinical Genetics Resource - a comprehensive clinically annotated database and resource for genetic variants in ATP7A gene. Comput. Struct. Biotechnol. J..

[bib15] Møller L.B. (2015). Small amounts of functional ATP7A protein permit mild phenotype. J. Trace Elem. Med. Biol. Organ Soc .Miner. Trace Elem. GMS.

[bib16] Møller L.B., Mogensen M., Weaver D.D., Pedersen P.A. Occipital horn syndrome as a result of splice site mutations in ATP7A. No activity of ATP7A splice variants missing exon 10 or exon 15. https://www.frontiersin.org/article/10.3389/fnmol.2021.532291.

[bib17] Nadal D., Baerlocher K. (1988). Menkes' disease: long-term treatment with copper and D-penicillamine. Eur. J. Pediatr..

[bib18] Rangarh P., Kohli N. (2018). Neuroimaging findings in Menkes disease: a rare neurodegenerative disorder. BMJ Case Rep..

[bib19] Shibuya M., Yaoita H., Kodama K., Okubo Y., Endo W., Inui T. (2022). A patient with early-onset SMAX3 and a novel variant of ATP7A. Brain Dev..

[bib20] Skjørringe T., Amstrup Pedersen P., Salling Thorborg S., Nissen P., Gourdon P., Birk Møller L. (2017). Characterization of ATP7A missense mutants suggests a correlation between intracellular trafficking and severity of Menkes disease. Sci. Rep..

[bib21] Sobczyńska-Tomaszewska A., Ołtarzewski M., Czerska K., Wertheim-Tysarowska K., Sands D., Walkowiak J. (2013). Newborn screening for cystic fibrosis: polish 4 years' experience with CFTR sequencing strategy. Eur. J. Hum. Genet. EJHG.

[bib22] Thomas H.B., Wood K.A., Buczek W.A., Gordon C.T., Pingault V., Attié-Bitach T. (2020). EFTUD2 missense variants disrupt protein function and splicing in mandibulofacial dysostosis Guion-Almeida type. Hum. Mutat..

[bib23] Tønnesen T., Kleijer W.J., Horn N. (1991). Incidence of Menkes disease. Hum. Genet..

[bib24] Trackman P.C. (2018). Functional importance of lysyl oxidase family propeptide regions. J. Cell Commun Signal.

[bib26] Tümer Z., Petris M., Zhu S., Mercer J., Bukrinski J., Bilz S. (2017). A 37-year-old Menkes disease patient-Residual ATP7A activity and early copper administration as key factors in beneficial treatment. Clin. Genet..

[bib27] Vairo FP e, Chwal B.C., Perini S., Ferreira M.A.P., de Freitas Lopes A.C., Saute J.A.M. (2019). A systematic review and evidence-based guideline for diagnosis and treatment of Menkes disease. Mol. Genet. Metabol..

[bib28] Vincent S., Robertson D. (2002). The broader view: catecholamine abnormalities. Clin. Auton. Res. Off. J. Clin. Auton. Res. Soc..

[bib29] Yasmeen S., Lund K., De Paepe A., De Bie S., Heiberg A., Silva J. (2014). Occipital horn syndrome and classical Menkes Syndrome caused by deep intronic mutations, leading to the activation of ATP7A pseudo-exon. Eur. J. Hum. Genet. EJHG.

[bib30] Zlatic S., Comstra H.S., Gokhale A., Petris M.J., Faundez V. (2015). Molecular basis of neurodegeneration and neurodevelopmental defects in Menkes disease. Neurobiol. Dis..

