# Supplementary material for: Deep intronic variant causes aberrant splicing of ATP7A in a family with a variable occipital horn syndrome phenotype
Source: Eur J Med Genet. 2024 Feb;67:104907. doi: 10.1016/j.ejmg.2023.104907 (PMC10918460; doi:10.1016/j.ejmg.2023.104907)
Supplement: Multimedia component 1 [file mmc1.docx]

| Chr | Start | End | Gene | No times seen | Comments |
| --- | --- | --- | --- | --- | --- |
| X | 334,257 | 334,388 | *PPP2R3B* | 8 |  |
| X | 1,497,621 | 1,497,711 | *IL3RA* | 1 | Pseudoautosomal region |
| X | 2,418,315 | 2,418,627 | *ZBED1* | 17 |  |
| X | 2,609,409 | 2,609,592 | *CD99* | 7 |  |
| X | 3,772,984 | 3,773,085 | *ENST00000456563* | 8 | In region |
| X | 8,699,931 | 8,700,103 | *KAL1* | 14 |  |
| X | 9,754,625 | 9,754,818 | *SHROOM2* | 5 |  |
| X | 17,393,878 | 17,394,444 | *NHS* | 10 |  |
| X | 19,533,285 | 19,533,411 | *MAP3K15* | 9 |  |
| X | 20,206,470 | 20,206,590 | *RPS6KA3* | 5 |  |
| X | 21,958,955 | 21,959,077 | *SMS* | 15 |  |
| X | 21,959,261 | 21,959,551 | *SMS* | 5 |  |
| X | 47,003,838 | 47,003,958 | *NDUFB11* | 12 |  |
| X | 49,020,098 | 49,020,422 | *MAGIX* | 4 |  |
| X | 49,772,917 | 49,773,037 | *CLCN5* | 1 | Within small shared region (0.6 Mb); chloride channel mutated in Dent disease (progressive proximal renal tubulopathy with hypercalciuria, low-molecular-weight proteinuria, and nephrocalcinosis). Not an exon on Alamut |
| X | 49,773,042 | 49,773,162 | *CLCN5* | 6 |  |
| X | 55,513,621 | 55,514,979 | *USP51* | 17 | In region |
| X | 57,618,454 | 57,618,710 | *ZXDB* | 5 | In region |
| X | 70,586,296 | 70,586,416 | *TAF1* | 9 | In region |
| X | 100,268,662 | 100,268,753 | *TRMT2B* | 9 |  |
| X | 101,093,015 | 101,093,203 | *NXF5* | 19 |  |
| X | 101,581,338 | 101,581,463 | *NXF2B* | 11 |  |
| X | 103,231,281 | 103,231,429 | *H2BFXP* | 15 |  |
| X | 103,231,489 | 103,231,579 | *H2BFXP* | 14 |  |
| Chr | Start | End | Gene | No times seen | Comments |
| X | 106,243,090 | 106,243,214 | *MORC4* | 15 |  |
| X | 115,568,919 | 115,569,154 | *SLC6A14* | 6 |  |
| X | 120,504,772 | 120,505,002 | *hsa-mir-3672* | 1 | Not within a shared region |
| X | 152,751,165 | 152,751,576 | *HAUS7* | 1 | Within a small shared region (0.74 Mb). Part of the mitotic spindle assembly. Not an exon on Alamut |
| X | 153,285,281 | 153,285,402 | *ENST00000369980* | 5 |  |
| X | 153,520,375 | 153,520,470 | *TEX28* | 22 |  |
| X | 153,872,142 | 153,872,280 | *ENST00000453062* | 7 |  |
